# Clusters of Adolescent Physical Activity Tracker Patterns and Their Associations With Physical Activity Behaviors in Finland and Ireland: Cross-Sectional Study

**DOI:** 10.2196/18509

**Published:** 2020-09-01

**Authors:** Kwok Ng, Sami Kokko, Tuija Tammelin, Jouni Kallio, Sarahjane Belton, Wesley O'Brien, Marie Murphy, Cormac Powell, Catherine Woods

**Affiliations:** 1 School of Educational Sciences and Psychology University of Eastern Finland Joensuu Finland; 2 Department of Physical Education and Sport Sciences University of Limerick Limerick Ireland; 3 Physical Activity for Health Cluster, Health Research Institute University of Limerick Limerick Ireland; 4 Faculty of Sport and Health Sciences University of Jyvaskyla Jyväskylä Finland; 5 LIKES Research Centre for Physical Activity and Health Jyväskylä Finland; 6 School of Health and Human Performance Faculty of Science and Health Dublin City University Dublin Ireland; 7 School of Education University College Cork Cork Ireland; 8 School of Sport Ulster University Belfast United Kingdom; 9 Performance Department Swim Ireland Irish Sport HQ Dublin Ireland

**Keywords:** wearables, children, activity trackers, active travel, organised sport, self-quantification

## Abstract

**Background:**

Physical activity trackers (PATs) such as apps and wearable devices (eg, sports watches, heart rate monitors) are increasingly being used by young adolescents. Despite the potential of PATs to help monitor and improve moderate-to-vigorous physical activity (MVPA) behaviors, there is a lack of research that confirms an association between PAT ownership or use and physical activity behaviors at the population level.

**Objective:**

The purpose of this study was to examine the ownership and use of PATs in youth and their associations with physical activity behaviors, including daily MVPA, sports club membership, and active travel, in 2 nationally representative samples of young adolescent males and females in Finland and Ireland.

**Methods:**

Comparable data were gathered in the 2018 Finnish School-aged Physical Activity (F-SPA 2018, n=3311) and the 2018 Irish Children’s Sport Participation and Physical Activity (CSPPA 2018, n=4797) studies. A cluster analysis was performed to obtain the patterns of PAT ownership and usage by adolescents (age, 11-15 years). Four similar clusters were identified across Finnish and Irish adolescents: (1) no PATs, (2) PAT owners, (3) app users, and (4) wearable device users. Adjusted binary logistic regression analyses were used to evaluate how PAT clusters were associated with physical activity behaviors, including daily MVPA, membership of sports clubs, and active travel, after stratification by gender.

**Results:**

The proportion of app ownership among Finnish adolescents (2038/3311, 61.6%) was almost double that of their Irish counterparts (1738/4797, 36.2%). Despite these differences, the clustering patterns of PATs were similar between the 2 countries. App users were more likely to take part in daily MVPA (males, odds ratio [OR] 1.27, 95% CI 1.04-1.55; females, OR 1.49, 95% CI 1.20-1.85) and be members of sports clubs (males, OR 1.37, 95% CI 1.15-1.62; females, OR 1.25, 95% CI 1.07-1.50) compared to the no PATs cluster, after adjusting for country, age, family affluence, and disabilities. These associations, after the same adjustments, were even stronger for wearable device users to participate in daily MVPA (males, OR 1.83, 95% CI 1.49-2.23; females, OR 2.25, 95% CI 1.80-2.82) and be members of sports clubs (males, OR 1.88, 95% CI 1.55-2.88; females, OR 2.07, 95% CI 1.71-2.52). Significant associations were observed between male users of wearable devices and taking part in active travel behavior (OR 1.39, 95% CI 1.04-1.86).

**Conclusions:**

Although Finnish adolescents report more ownership of PATs than Irish adolescents, the patterns of use and ownership remain similar among the cohorts. The findings of our study show that physical activity behaviors were positively associated with wearable device users and app users. These findings were similar between males and females. Given the cross-sectional nature of this data, the relationship between using apps or wearable devices and enhancing physical activity behaviors requires further investigation.

## Introduction

### Background

Physical inactivity is one of the leading causes of worldwide mortality. There is an urgent need to understand how to increase physical activity levels among young adolescents (typically aged between 11 years and 15 years). The habits developed during early adolescence will continue through adulthood [1], particularly for physical activity, in both the short-term [2,3] and long-term [4,5]. Although technologies have evolved in the last generation, less is known about the how the use of physical activity trackers (PATs) in young adolescents can be used to understand their physical activity behaviors. Lee et al [6] conducted a scoping review and included 14 studies that included intervention components such as websites, apps, and wearables devices. Apps and wearable devices include the interaction of sensors that have the capability to measure physical activity, and for the purpose of this study, they are referred throughout as PATs. Small to medium effect sizes in adult studies have been demonstrated, specifically when individuals used the PAT information to modify their behaviors and increase their physical activity levels [7]; however, intervention studies among adolescents are rare [6]. The lesser number of studies on adolescents may be attributed to the way apps and wearable devices have been designed—they are primarily designed with the adult population in mind, thereby leading to low levels of use among adolescents [8]. Even though there have been recent developments in companies to build PATs for youngsters, the feedback from children through their parents still indicate that design issues are present, which can cause a barrier for sustainable use [9]. Understanding the association between PAT ownership and use may help inform their use as an effective intervention tool in the young adolescent population.

### Country-Specific Usage

The use of apps requires the use of smartphones and consistent internet connectivity. In Ireland, the prevalence of mobile phone use among 13-year-old adolescents has been reported to be 98% [10], with 54% reporting to use the internet with their phones [11]. In Finland, 93% of the adolescents aged 16 years use their mobile phones to access the internet and this has been facilitated by the way mobile phone subscription plans in Finland typically offer unlimited data [12]. According to Eurostat, 94% of the homes in Finland have access to the internet, whereas, in Ireland, internet household accessibility stands at 89%, which is equivalent to the average in European Union countries [13].

In Finland, wearable devices are used in 22% of the households [12], but the use of wearable devices in Ireland has not been reported. Younger generations often report greater use of the internet than the average population [14], and this information is frequently used to ensure that PAT functionality is optimal for age-appropriate usage.

Both countries perform highly in terms of progression in making societies mediated by digital technology. For examples, out of all the OECD (Organization for Economic Cooperation and Development) countries, Finns use the most amount of mobile data per subscriber (OECD, 2018), and Finland has been ranked the highest for digital services in all of Europe [15]. Ireland, on the other hand, is the second ranked country in Europe, outside of the Nordic countries, in the digital economy and society index [13]. One of the major differences between Finland and Ireland is the human capital. Ireland is ranked number 1 in terms of open data usage; however, it is placed 21st in the European Union for eHealth services, with just 1 in 10 people (11%) using it. However, in Finland, open data usage tops the eHealth services index, with 49% of the individuals using it [15].

### PATs for Facilitation of Physical Activity Behaviors

Emerging evidence suggests that PATs have a positive effect on physical activity behaviors, particularly as facilitators, rather than as drivers of health behavior change [16]. From the end user’s perspective, one significant limitation of commercial PAT products is the short life cycle [17,18]. It has been estimated that 10% of the global population use fitness apps, but only 2% are paying users [19]. These figures have increased in the last few years and are expected to increase by 4.5% every year at least until 2024 [19]. Despite the commercial growth in the market, the expected level of success of PAT ownership in terms of physical activity behavior change is yet to be fully interrogated or proven. Most of the functions within apps include between 5 and 8 behavioral change techniques, with common features including goals and planning, feedback and monitoring, social support and shaping behavior [20]. Several trials have investigated the efficacy of PATs in increasing physical activity levels, and often, although an initial increase in the physical activity level is documented, these changes are not sustained in the long term [18,21,22] or could not be replicated with younger populations [23]. High attrition levels are particularly common for PATs, where the novelty effect wears off and so does the usage and effectiveness [24]. Designers of PATs could be partly responsible for this attrition, as the products made may not be sufficiently accurate enough to meet the demands of the end user [25,26] or the automated systems for providing reminders and feedback are deemed inadequate [27]. The majority of PAT features include functions that can be used for socializing between users, which is important for young adolescent populations [9]. Moreover, young adolescents see the benefits of multipurpose devices as this can help individuals track various aspects of their lives such as health [28]. Despite this, young adolescents have reported that PATs currently do not provide enough customization or personalization [8].

Differences have been observed in the way that males and females use the multiple functions of PATs. For example, male adolescents prefer to socialize with their PATs through “banter” and other friendly conversations [29]. Female PAT users report lower levels of aspirations to take part in competitive sports than male PAT users [30], which may translate into less use of the goal and planning functions [31]—the areas commonly used for optimal performance in sports [32]. Feedback from PATs is also central to the control theory [33], as it confirms user performance to prompt further behaviors. For the purpose of this study, we focused on the association between PAT ownership and use and 3 behaviors, namely, (1) overall physical activity behavior, (2) participation in sports clubs, and (3) active commuting. Research on PATs as an intervention for adolescents is limited [8], and to our knowledge, only a handful of observational studies have been published on PAT usage in young adolescents [34]. Therefore, the purposes of this study were to investigate the differences in PAT ownership and usage between Finnish and Irish adolescents, while investigating the association between PAT ownership and self-reported physical activity behaviors in both cohorts and considering the potential influence of gender.

## Methods

### Recruitment

Data for this cross-sectional study were collected in Finland and Ireland during the first half of 2018. Both the Finnish and Irish data were collected from national representative cross-sectional studies. In Finland, the Finnish School-aged Physical Activity (F-SPA) study [35] is the national physical activity monitoring study for children and adolescents (LIITU in Finnish) and the Irish equivalent is the Children’s Sport Participation and Physical Activity (CSPPA) study [36].

The F-SPA 2018 was based on 2-level cluster analyses [35] and is an extension to the F-SPA 2014 and F-SPA 2016 studies by including students as young as 7 years of age. A parallel study was conducted for special education classes and schools; hence, sampling did not include special education schools. The probability proportion size was used to calculate the primary sampling unit to generate a nationally representative sample. This study consisted of a total of 311 schools (Finnish schools: 267/311, 85.8%; Swedish schools: 44/311, 14.1%) in Finland and 9940 students altogether.

The CSPPA 2018 was a follow-up and extension to the original 2010 study [37]. CSPPA 2018 included schools from both the Republic of Ireland and Northern Ireland. The sampling frame for the schools involved in CSPPA 2010 included all schools with students aged between 10 years and 18 years in the Republic of Ireland. A systematic one-stage cluster sampling method was used for CSPPA 2010 and schools were stratified by 4 criteria (school gender, school socioeconomic status, school location, and school size). The same schools in 2010 were invited again to take part in 2018, and a replacement list based on the creation of an equivalence sample was made to ensure that sufficient number of students were included to allow for study design effects. Schools from Northern Ireland were not part of CSPPA 2010 and all mainstream schools from Northern Ireland were included in the sampling frame for CSPPA 2018. Special schools, primary schools, and colleges of further education were excluded from the database. In total, in the Republic of Ireland and Northern Ireland, 115 schools were included in the overall study sample and 6651 students took part in CSPPA 2018.

All surveys were completed on either a tablet, laptop, or personal computer in the students’ own classroom and under the supervision of teachers in F-SPA 2018 or specifically trained research assistants in CSPPA 2018. Students who were given permissions by their parents or guardians had the right to withdraw from the study at any time. The completion of the survey was done anonymously and voluntarily. The Finnish study was approved by the ethics committee of the University of Jyvaskyla, Finland, where no number was provided, and the Irish study was approved by the ethics committee of the University of Limerick, Ireland.

For the purpose of comparisons between the studies, only responses from young adolescents aged between 11 years and 15 years were included (Finland, n=3311; Ireland, n=4797) in the final data set. Variables for the country data files were relabeled to allow for merging in SPSS 25.0 (IBM Corp). The details of the measures in both surveys used for this study are reported in the table in Multimedia Appendix 1.

### Measures

Both surveys collected demographic information on gender, age, disability status, and self-reported socioeconomic status via the Family Affluence Scale (FAS) [38]. The CSPPA 2018 study also included the option of “other” to respond for gender, whereas the F-SPA 2018 did not; therefore, respondents with “other” (69/6649, 1.0%) were removed from the CSPPA 2018 sample prior to cleaning the data file for analyses.

Items of PATs had slight variation (Multimedia Appendix 1). In Finland, a block question was designed to keep the survey length to the minimum. The opening question was, “How often in a week do you use the following physical activity tracking devices?” with the following options, “mobile apps,” “activity meter or sports watch,” and “heart rate monitor.” This item was used in the previous edition of the Finnish monitoring study [34]. Although the response options were updated, based on the feedback from the test-retest reliability study [39], the previous 3 categories of “none,” “own but do not use”, and “own and use” were extended to a 6-item category frequency scale of (1) I don’t have, (2) Never, (3) At least once a week, (4) Once a day, (5) More than once a day, and (6) All the time. The variables were divided into 3 groups to match with the previous reporting, whereby respondents to 1 were grouped as “do not have,” 2 were grouped as “own but do not use,” and 3 to 6 were grouped as “own and use.”

The Irish version had separate questions on ownership, use, and frequency of use for (1) physical activity apps, (2) smartwatches, (3) heart rate monitors, (4) pedometers, and (5) other devices. For comparison purposes, individuals who responded to only having a pedometer or other device (528/4797; 11.0%) were recoded as not having a PAT since this was not compatible between the 2 studies. There was a slight variation in the frequency of use of the PATs, because the question was, “How often do you use your physical activity tracking device during a typical week” with response options (1) Never, (2) Once, (3) Sometimes, (4) Almost every day, and (5) Every day. Responses of Never were grouped into “own but do not use” and 2-5 were grouped into “own and use.” Null responses to the ownership were deemed as “do not have.”

The other survey responses used included the self-reported number of days of at least 60 minutes of moderate-to-vigorous physical activity (MVPA) participation in both 2018 F-SPA and CSPPA studies. The CSPPA 2018 study included 2 items based on the past 7 days and the usual week. The 2 items were summed and divided by 2 and rounded up, whereas the F-SPA 2018 item included 1 item based on the past 7 days. Previous studies suggest that an average between the previous week and the usual week can provide a more accurate recall of physical activity behaviors [40]; however, in other international surveys, a single item was used to reduce the number of items in the survey [41]. Both surveys used the same explanation to define MVPA, as comparable to an international study protocol [42]. The physical activity survey item was then dichotomized into meeting the guidelines (specifically when the respondents reported a total of 7 days) and not meeting the guidelines (specifically when the respondents reported anything between 0 and 6 days). This physical activity survey item has been previously tested for validity use against accelerometers with young adolescents [40,43] and within test-retest environments [44,45].

Respondents provided details of their mode of transport to school with walking or cycling categorized as “active commuters.” Motorized transport included options such as getting a lift by parents or taking the bus. The distance between the primary home and school was also asked. To ensure that the distances were plausible for active transport, the Finnish legislation for the provision of free transport costs were set at distances over 5 km as the cut-off point to differentiate between people who were close (within 5 km) and far (over 5 km). For the inferential statistics regarding active commuting, only respondents who lived within the close range (5 km) of the school were included. Therefore, living beyond 5 km was an exclusion criterion for the analyses in relation to active commuting.

### Statistical Analysis

Descriptive statistics of the population characteristics were produced by chi-square tests of independence for gender, after stratifying for country. The test of independence between the countries was also tested through chi-square tests after considering the gender. A two-step approach was used to describe the phenomenon of PAT ownership and use among young adolescents in Finland and Ireland. The number of possible combinations of PAT habits was investigated using cluster analysis to the fewest number of clusters, yet attempting to retain a meaningful structure (ie, values of the average silhouette width defining the cluster quality as “good” [exceeding 0.5]) [46]. When the sample was pooled, 3 clusters were deemed to be sufficient; however, when the test of clusters was examined for each country, one of the clusters in each country had different features; therefore, an extra cluster was added. The characteristics of the 4 clusters were then tested in the pooled sample, and individually, each country achieved good cluster quality (Finland silhouette width=0.6, Ireland silhouette width=0.7) and led to 4 clusters being identified. The first cluster (and reference category for regression analyses) included individuals who reported no ownership of apps, smartwatches, or heart rate monitors. This category was labelled as “no PATs.” The second cluster predominately included individuals who reported ownership but not usage of PATs and were labelled as “PAT owners.” The third cluster was labelled as “app users” as the majority of the app users belonged to this cluster, with none in the cluster reporting the use of smartwatches or heart rate monitors. The fourth cluster included a mixture of individuals consistently using smartwatches and heart rate monitors and they were subsequently labelled as “wearable device users.”

Chi-square tests were used to assess the statistical significance of gender, age groups, FAS, and disability for the clusters, and the Kruskal-Wallis test with pairwise comparisons was used to assess the statistical significance of the differences in the average number of days reporting 60 minutes of MVPA for each country.

The binary logistic associations of meeting the physical activity guidelines (7 days vs <7 days, reference category), being an active traveler (cyclist and walker vs motorized transport who live within 5 km of the school, reference category), and organized sport participant (sports club member vs not active in sports clubs, reference category) with no ownership of PATs as the reference category were investigated. The crude associations for each indicator (Model 1) were assessed before adjusting for age, gender, FAS, and disability (Model 2). All statistics were run using SPSS 25.0 for Windows (released 2017).

## Results

### User Statistics

The descriptive statistics are provided in Table 1 with comparisons between and within countries.

**Table 1:** Descriptive statistics of the samples by country and gender.

Characteristics		Finland (n=3311)						Ireland (n=4797)						Total^a^ (N=8108)					
		Males, n= 1610, n (%)		Females, n=1701, n (%)		*P* value		Males, n=2370, n (%)		Females, n=2427, n (%)		*P* value		Males, n= 3980, n (%)		Females, n= 4128, n (%)		*P* value	
**Age (years)**						.81						.003						.01	
	11		628 (39.0)		680 (39.9)				402 (16.9)		468 (19.3)				1030 (25.9)		1148 (27.8)		
	13		452 (28.1)		477 (28.0)				1090 (45.9)		1168 (48.1)				1542 (38.7)		1645 (39.8)		
	15		530 (32.9)		544 (31.9)				878 (37.0)		791 (32.6)				1408 (35.4)		1335 (32.3)		
**FAS^b^**						.81						.05						.09	
	Low		357 (24.8)		374 (24.4)				495 (20.9)		529 (21.8)				852 (22.4)		903 (22.8)		
	Middle		827 (57.4)		874 (56.9)				1398 (58.9)		1351 (55.7)				2225 (58.4)		2225 (56.2)		
	High		256 (17.8)		287 (18.7)				477 (20.1)		546 (22.5)				733 (19.2)		833 (20.2)		
**Disability**						.07						.66						.17	
	None		1439 (89.9)		1493 (87.8)				2035 (85.9)		2073 (85.4)				3474 (87.5)		3566 (86.5)		
	Disabled		161 (10.06)		204 (11.9)				335 (14.1)		354 (14.6)				496 (12.5)		558 (13.5)		
**Daily MVPA^c^**						<.001						<.001						<.001	
	Inactive^d^		1041 (64.8)		1211 (71.3)				1955 (82.5)		2157 (88.9)				2996 (75.3)		3368 (81.6)		
	Active^e^		566 (35.2)		488 (28.7)				415 (17.5)		270 (11.1)				981 (24.7)		758 (18.4)		
**Transport**						.15						<.001						.02	
	Motorized, Close		213 (13.5)		213 (12.7)				504 (28.7)		583 (33.1)				717 (21.5)		796 (23.1)		
	Active, Close		916 (58.1)		1005 (59.8)				430 (24.5)		335 (19)				1346 (40.4)		1340 (39)		
	Active, Far		80 (5.07)		60 (3.6)				44 (2.5)		30 (1.7)				124 (3.7)		90 (2.6)		
	Motorized, Far		368 (23.3)		403 (24)				779 (44.3)		811 (46.1)				1147 (34.4)		1214 (35.3)		
**Sports club**						.64						.04						.06	
	Nonmember		629 (40.5)		687 (41.31)				852 (35.95)		941 (38.77)				1481 (38.8)		1628 (39.8)		
	Member		924 (59.5)		976 (58.69)				1518 (64.05)		1486 (61.23)				2442 (61.2)		2462 (60.2)		
**Apps**						<.001						<.001						<.001	
	Not owned		624 (38.7)		649 (38.2)				1602 (67.6)		1457 (60)				2226 (55.9)		2106 (51.0)		
	Do not use		342 (21.2)		244 (14.3)				274 (11.6)		347 (14.3)				616 (15.5)		591 (14.3)		
	Use		644 (40)		808 (47.5)				494 (20.8)		623 (25.7)				1138 (28.6)		1431 (34.7)		
**Sports watch**						<.001						.56						<.001	
	Not owned		1080 (68.6)		1240 (74.1)				1836 (77.5)		1903 (78.4)				2916 (73.9)		3143 (76.6)		
	Do not use		269 (17.1)		182 (10.9)				169 (7.1)		155 (6.4)				438 (11.0)		337 (8.2)		
	Use		225 (14.3)		252 (15.1)				365 (15.4)		369 (15.2)				590 (15.0)		621 (15.1)		
**Heart rate monitor**						<.001						.19						<.001	
	Not owned		1119 (71.4)		1331 (79.6)				2146 (90.5)		2230 (91.9)				3265 (82.9)		3561 (86.9)		
	Do not use		285 (18.2)		201 (12)				55 (2.3)		55 (2.3)				340 (8.6)		256 (6.2)		
	Use		164 (10.5)		141 (8.4)				169 (7.1)		142 (5.9)				333 (8.5)		283 (6.9)		

^a^The percentages in this column are the actual percentages and not of the total population because some data on the variables of the total population were missing.

^b^FAS: Family Affluence Scale.

^c^MVPA: moderate-to-vigorous physical activity.

^d^0-6 days of MVPA.

^e^7 days of MVPA.

There were statistical differences between the characteristics of Finnish and Irish young adolescents between the 2 surveys. The CSPPA 2018 study had fewer 11-year-old adolescents than those in the F-SPA 2018, as the participants were more evenly distributed across the varying age groups in the F-SPA 2018 (chi-square *P*=.75). There were fewer Finnish (n=3311) than Irish (n=4797) respondents in the final sample (Table 1).

The estimates of ownership and usage of apps (*P*<.001), sports watches (*P*<.001), and heart rate monitors (*P*<.001) were significantly different between Finnish and Irish adolescents. In Finland, almost two-thirds (2038/3311, 61.6%) of the young adolescents reported owning or using apps to monitor physical activity, whereas in Ireland, the majority (3059/4797, 63.8%) did not own apps to monitor physical activity. Over three-quarter of the Irish adolescents do not own sports watches and this was a greater estimate than that among Finnish adolescents (2320/3311, 71.4%; *P*<.001). Moreover, a quarter (791/3311, 24.4%) of the Finnish adolescents reported owning or using heart rate monitor, which was much higher than that in Ireland (421/4797, 8.8%; *P*<.001).

### Cluster Analyses

The 4 clusters were ”no PATs,” “PAT owners,” “app users,” and “wearable device users” (Table 2), with a silhouette of 0.7 for cluster quality exceeding the good threshold of 0.5 [46]. The crude percentage of the individuals who reported daily MVPA was almost double among wearable device users (498/1631, 30.6%) compared to those in no PATs (576/3523, 16.3%). The estimates of those involved in active transport from cluster 1 (1033/1732, 59.6%), cluster 2 (236/373, 63.3%), cluster 3 (804/1217, 66.1%), and cluster 4 (571/826, 69.1%) and of sports clubs members (cluster 1, 1957/3523, 56.2%; cluster 2, 399/677, 59.6%; cluster 3, 1333/2200, 61.4%; and cluster 4, 1178/1631, 73.1%) were high.

**Table 2:** Features of the four clusters from pooled data and crude estimates of the behaviors.

Features		Cluster 1 (No PATs), n=3523, n (%)		Cluster 2 (PAT owners), n=677, n (%)		Cluster 3 (app users), n=2200, n (%)		Cluster 4 (wearable device users), n=1631, n (%)	
**Apps**									
	None		3523 (100.0)		265 (39.1)		0 (0)		531 (32.6)
	Own		0 (0)		412 (60.9)		701 (31.9)		85 (5.2)
	Use		0 (0)		0 (0)		1499 (68.1)		1015 (62.2)
**Smartwatches**									
	None		3523 (100.0)		72 (10.6)		2200 (100)		256 (15.7)
	Own		0 (0)		605 (89.4)		0 (0)		167 (10.2)
	Use		0 (0)		0 (0)		0 (0)		1208 (74.1)
**Heart rate monitors**									
	None		3523 (100.0)		286 (42.2)		2200 (100)		814 (49.9)
	Own		0 (0)		391 (57.8)		0 (0)		204 (12.5)
	Use		0 (0)		0 (0)		0 (0)		613 (37.6)
**Crude percentage**									
	Daily MVPA^a^		576 (16.3)		130 (19.2)		503 (22.9)		498 (30.6)
	Active Transport^b^		1033^c^ (59.6)		236^d^ (63.3)		804^e^ (66.1)		571^f^ (69.1)
	Sports club		1957 (56.2)		399 (59.6)		1333 (61.4)		1178 (73.1)

^a^MVPA: moderate-to-vigorous physical activity.

^b^Fewer people in this subcategory met the criteria of living within 5 km; therefore, the sample population for this row is different in each cluster as shown in the following footnotes.

^c^n=1732.

^d^n=373.

^e^n=1217.

^f^n=826.

### Multivariate Analyses of the Males

In the unadjusted model (Model 1, Table 3), daily MVPA (odds ratio [OR] 1.56, 95% CI 1.29-1.87; OR 2.16, 95% CI 1.79-2.60), active transport (OR 1.41, 95% CI 1.13-1.77; OR 1.83, 95% CI 1.41-2.36), and being members of sports clubs (OR 1.32, 95% CI 1.12-1.56; OR 1.97, 95% CI 1.64-2.36) showed positive associations with male app users and wearable device users, respectively. Moreover, owners of PATs were positively associated with active travel (OR 1.40, 95% CI 1.02-1.91). After controlling for country, age, FAS, and disabilities, the associations had lower ORs and the association for active travel and app users was no longer statistically significant (Model 2, Table 3). Disabilities were also not associated with active travel, whereas as adolescents became older, the less likely they would be involved in physical activity behaviors. Higher FAS was positively associated with daily MVPA and being a member of sports clubs, whereas it was negatively associated with active travel. There were more Irish adolescents who were members of sports clubs, but significantly fewer who took part in daily MVPA or active travel.

**Table 3:** Male-adjusted odds ratios and 95% confidence intervals without Model 1 and with Model 2 confounders for each cluster. Italics represents statistically significant associations.

Variables			Moderate-to-vigorous physical activity^a^, OR^b^ (95% CI)	Active travel^c^, OR (95% CI)	Sports club^d^, OR (95% CI)
**Model 1**					
	No PATs		Reference (1.0)	Reference (1.0)	Reference (1.0)
	PAT owners		1.17 (0.89-1.53)	*1.40 (1.02-1.91)*	1.03 (0.82-1.29)
	App users		*1.56 (1.29-1.87)*	*1.41 (1.13-1.77)*	*1.32 (1.12-1.56)*
	Wearable device users		*2.16 (1.79-2.60)*	*1.83 (1.41-2.36)*	*1.97 (1.64-2.36)*
**Model 2**					
	No PATs		Reference (1.0)	Reference (1.0)	Reference (1.0)
	PAT owners		1.07 (0.81-1.42)	1.19 (0.84-1.69)	1.08 (0.85-1.37)
	App users		*1.27 (1.04-1.55)*	1.06 (0.82-1.36)	*1.37 (1.15-1.62)*
	Wearable device users		*1.83 (1.49-2.23)*	*1.39 (1.04-1.86)*	*1.88 (1.55-2.28)*
	**Country**				
		Finland	Reference (1.0)	Reference (1.0)	Reference (1.0)
		Ireland	*0.46 (0.39-0.54)*	*0.26 (0.21-0.32)*	*1.44 (1.25-1.67)*
	**Age**				
		Young	Reference (1.0)	Reference (1.0)	Reference (1.0)
		Older	*0.69 (0.63-0.77)*	*0.59 (0.50-0.69)*	*0.68 (0.62-0.74)*
	**Family Affluence Scale**				
		Lower	Reference (1.0)	Reference (1.0)	Reference (1.0)
		Higher	*1.22 (1.08-1.37)*	*0.78 (0.66-0.92)*	*1.39 (1.23-1.55)*
	**Disability**				
		Without	Reference (1.0)	Reference (1.0)	Reference (1.0)
		With	*0.59 (0.45-0.77)*	1.00 (0.74-1.40)	*0.60 (0.49-0.73)*

^a^Reference=not daily, Nagelkerke *R^2^* (Model 1)=0.026, Nagelkerke *R^2^* (Model 2)=0.102.

^b^OR: odds ratio.

^c^Reference=motorized, Nagelkerke *R^2^* (Model 1)= 0.017, Nagelkerke *R^2^* (Model 2)=0.217.

^d^Reference=not member, Nagelkerke *R^2^* (Model 1)=0.020, Nagelkerke *R^2^* (Model 2)=0.075.

### Multivariate Analyses of the Females

In the unadjusted model (Model 3, Table 4), wearable device users were twice as likely to report MVPA (OR 2.45, 95% CI 2.00-3.02) and be member of organized sports (OR 2.29, 95% CI 1.91-2.74). The associations were not as strong for app users, and for active travel, the association was similar between app users (OR 1.24, 95% CI=1.01-1.53) and wearable device users (OR 1.28, 95% CI 1.01-1.63). Owners of PATs were more likely to be members of sports clubs (OR 1.31, 95% CI 1.01-1.68) when compared to the no PATs cluster. After controlling for country, age, FAS, and disabilities (Model 4, Table 4), the ORs were lower, but the model strengths were stronger. There were no significant differences between no ownership or usage of PATs and any cluster of PATs. Females with and without disabilities were not different in terms of MVPA and active travel.

**Table 4:** Female-adjusted odds ratios and 95% confidence intervals without Model 3 and with Model 4 confounders for each cluster. Italics represents statistically significant associations.

Variables			Moderate-to-vigorous physical activity^a^, OR^b^ (95% CI)	Active travel^c^, OR (95% CI)	Sports club^d^, OR (95% CI)
**Model 3**					
	None		Reference (1.0)	Reference (1.0)	Reference (1.0)
	Owners		1.21 (0.86-1.72)	0.92 (0.65-1.30)	*1.31 (1.01-1.68)*
	App user		*1.61 (1.32-1.96)*	*1.24 (1.01-1.53)*	*1.21(1.04-1.40)*
	Wearable device user		*2.45 (2.00-3.02)*	*1.28 (1.01-1.63)*	*(2.29 (1.91-2.74)*
**Model 4**					
	None		Reference (1.0)	Reference (1.0)	Reference (1.0)
	Owners		1.27 (0.87-1.84)	1.14 (0.76-1.72)	1.27 (0.97-1.66)
	App user		*1.49 (1.20-1.85)*	0.98 (0.76-1.25)	*1.25 (1.07-1.50)*
	Wearable device user		*2.25 (1.80-2.82)*	0.91 (0,68-1.21)	*2.07 (1.71-2.52)*
	**Country**				
		Finland	Reference (1.0)	Reference (1.0)	Reference (1.0)
		Ireland	*0.38 (0.31-0.45)*	*0.16 (0.13-0.20)*	*1.35 (1.17-1.55)*
	**Age**				
		Young	Reference (1.0)	Reference (1.0)	Reference (1.0)
		Older	*0.55 (0.49-0.62)*	*0.50 (0.43-0.59)*	*0.62 (0.57-0.68)*
	**Family Affluence Scale**				
		Lower	Reference (1.0)	Reference (1.0)	Reference (1.0)
		Higher	*1.19 (1.04-1.36)*	*0.85 (0.72-0.99)*	*1.57 (1.42-1.75)*
	**Disability**				
		Without	Reference (1.0)	Reference (1.0)	Reference (1.0)
		With	1.00 (0.76-1.31)	1.19 (0.89-1.58)	*0.59 (0.48-0.71)*

^a^Reference=not daily, Nagelkerke *R^2^* (Model 3)=0.030, Nagelkerke *R^2^* (Model 4)=0.141.

^b^OR: odds ratio.

^c^Reference=motorized, Nagelkerke *R^2^* (Model 3)=0.005, Nagelkerke *R^2^* (Model 4)=0.320.

^d^Reference=not member, Nagelkerke *R^2^* (Model 3)=0.028, Nagelkerke *R^2^* (Model 4)=0.109.

## Discussion

### Principal Results

Apps were owned by approximately two-thirds of the Finnish adolescents and by one-third of the Irish adolescents, with more females in both countries owning apps than males. The estimates of sports watch ownership or use is 28.6% (928/3311) among young Finns and 22.1% (1058/4797) among young Irish adolescents. Approximately 9.2% (305/3311) of the Finnish adolescents and 6.5% (311/4797) of the Irish adolescents use heart rate monitors. Despite these differences, the clustering patterns of PATs were similar between both the countries.

Four cluster patterns for PATs were identified: (1) no PATs, (2) PAT owners, (3) app users, and (4) wearable device users. Compared to individuals in the no PATs cluster, wearable device users had stronger association with physical activity behaviors (daily MVPA, sports club member, active travel). The likelihood of taking part in daily MVPA, being a member of a sports club, or travelling to school by foot or bike among females was higher than that in males, thereby indicating strong positive associations between PAT usage and physical activity behaviors.

More males than females reported meeting the physical activity guidelines of daily MVPA for at least 60 minutes per day [47]. Moreover, approximately twice as many males and two-and-a-half times as many females in Finland reported meeting the daily MVPA guidelines (ie, ≥60 minutes) compared to Irish males and females. Finland tends to perform better than Ireland in studies that report international comparisons of MVPA [48], thereby indicating that the results of this study aligns with other international level findings. Over two and three times as many Finnish males and females, respectively, take part in active travel to school compared to Irish adolescents. The differences in the membership of sports clubs were not statistically significant between the adolescents in the 2 countries. According to the results of the 2016 Global Matrix on physical activity, there were similar differences in the grades between the Finland and Ireland [49].

The majority of the reports from the adult surveys suggest that there are similarities in the use of PATs between Finland and Ireland [50,51]. However, in this study, there were significantly more Finnish adolescents who owned or used PATs compared with their Irish counterparts. In 2016, approximately 53% of the Finnish adolescents aged between 11 years and 15 years reported to own apps [34]; however, this study (2018 data) shows that the rate of app ownership increased to approximately 62%, which is a 9% increase in a 2-year period. There was also a notable increase in the ownership of sports watches and heart rate monitors between 2016 and 2018, and this has followed the rate of market penetration in the last 2 years [19]. This is the first time that PAT figures from Irish adolescents have been reported, and data suggest that adolescents reported a higher rate of ownership of apps (36.2%) when compared to an Irish 18-24-year-old cohort (20%) [50]. Based on these growth figures, it may be viable to design interventions using physical activity apps in Finland currently and in the near future, whereas in Ireland, more adoption is needed for natural experiments or trials to take place; otherwise, interventions would need to control the novelty effect of introducing a new wearable device [24].

### Comparison With Prior Work

#### PATs and Daily MVPA

Both male and female users of apps and wearable devices had positive associations with daily MVPA compared to adolescents with no PATs. However, the physical activity behaviors of young adolescents who merely own but reported to not use PATs were not statistically different from that of individuals in the no PATs cluster. Some of the underlying reasons for these results can be related to the ownership and usage of PATs as a proxy for readiness for the behavior [52]. The functions available in PATs can enable regular monitoring [53], self-comparison of previous performances, and setting targets for current performances [9], and these are all deemed to be effective behavioral change techniques, as reported in previous studies [20]. As such, the feedback from PATs to the individual can be of educational benefit to the user [54]. The details of the habits in using PATs need to be explored further to understand the mechanisms of these associations, particularly as individuals introduced to PATs in interventions experience attrition [17], thus limiting the sustainability of behavior change.

#### Use of PATs in Organized Sport

Similar to the users’ associations with MVPA, app users and wearable device users were more likely to report memberships in sports clubs, when compared to individuals with no PATs. Depending on the features and functionality of the specific PATs that individuals use, young adolescents can share data with other members of the sports club. This may increase motivation among males, as males are known to boast about their achievements with their peers [29]. Moreover, PATs could be used to support coaching practices by providing individualized information on athletes’ performance. Data have been used, wherein feedback on the physiological parameters such as heart rate can be informative to athletes and could reduce overtraining and thus the risk to injuries [55]. This is a promising area of development within wearable technology, wherein safety promotion and injury prevention features are built in. Seshadri and colleagues [56] further suggest that noninvasive sensors around the body such as earrings, headphones, rings, or articles made within textiles can act as a crucial pieces of technology to reduce the risk of injuries. Gabbett [57] argued that training harder can be protective of injuries if done smartly and if assisted through trackable data. This may be an important message for the general adolescent population, where health promoters aim to increase the levels of physical activity through careful planning to build up physical fitness. Not only do individuals have feedback on their own behavior, as postulated by the control theory, but the ideas and the information for taking part in more physical activities can be supported by the environment of sports clubs—typically the youth sports coach.

#### Active Travel and PATs

After adjusting for country, age, family affluence, and disabilities, the only significant association observed was between the male users of wearable devices and active travel. Although there are studies that suggest that PATs can help support more walking [21,27], previous studies have been based primarily on adults and the active travel behaviors of young adolescents are known to be heavily influenced by their parents [58]. Furthermore, distances between home and school that were considered as too far were over 4 km (2.5 miles) in Ireland [59] and 5 km or more away from the school in Finland, which resulted in a large reduction in the number of active commuters among young Finnish adolescents [60]. None of the adolescents in this study could legally use their own independent motorized transport by the age of 15 years, and yet, the active transport behavior was extremely different between Finland and Ireland. These national differences have been previously reported in the Global Matrix 2.0 physical activity report card, where Finland was graded “B” and Ireland as “D” [49].

Research on active travel is limited in terms of PATs; however, there have been some initiatives to promote active travel directly or indirectly through gamification [61,62]. These programs may start off well as the excitement of gamification kicks in, but later, the novelty can wear off, thereby reducing the potential to have sustained active travel [24]. Attrition may be avoided if designers of PATs use an established framework for functionalities in apps [63] and follow the principles around design and usage, as outlined by Attig and Franke [17].

Other initiatives for promoting active school travel and physical activity in general in schools may be created by using step challenges [54]. For young adolescents, such activities need to be considered with care. There could be negative effects [64] because some students have reported that they feel such challenges are impossible to win, given the head start others have on them if they started early in the morning. Alternatively, young adolescents have the feeling of guilt for not keeping up with the pace of their friends, as Goodyear and colleagues termed as “peer surveillance” [54].

Other innovative ways to increase active transport require the combination of technology with the Internet of Things, relying upon multiple sensors such as gyroscopes, GPS, and connectivity sensors so that students can interact more with each other [65]. In a previous study, children took part in a design session and identified that a light backpack and track commuting with their friends provided more opportunities to socialize [66]; this is an example of going beyond the concept of PATs for the purpose of tracking physical activity, but these can be used as a tool to engage with peers. The concept of wearables on bags is not a new idea, as the concepts have been considered already in the early part of the millennium [67], but it seems that commercial companies have been slow to convert this into the market.

### Theoretical Perspectives

Despite the differences in the levels of ownership and usage of PATs, this study found similarities in the clusters between Finnish and Irish adolescents. One of the limitations of the cluster analysis is the data-driven approach, which may lack representativeness outside of the population studied [68]. However, it is a recognized approach to investigate hierarchies and commonalities among groups [69]. It is likely that the difference between the 4 cluster groups is a combination of readiness for behavioral change [70] as well as a personal investment for self-quantification purposes [33,71]. The majority of the apps are free and can perform many of the same tasks, as what wearables can offer in terms of measuring the minimum level of physical activity for health, although it should be noted that the majority of the currently available PATs have been designed with the adult user in mind. Even with the latest models designed for children, the functions of PATs could be better improved to meet the needs of the young users [9]. Central to the sustainable use of PATs is the way in which feedback is given to the user. According to the control theory [33], a feedback loop is introduced between the motives and the behavior. As more wearable devices become available, this can form a part of the identity of an individual or be worn as a fashion item such as jewelry [65], which may be more appealing to females. Stronger beliefs can be seen through stronger commitment to a behavior [72]. Therefore, further research may be needed in the areas of clustering PAT ownership and usage with the role to maintain physical activity behaviors.

### Covariates of Associations

In both Finland and Ireland, there is a clear association between affluence and frequency in taking part in organized sports [11,73], as demonstrated in the fully adjusted model 2 and 4 for males and females, respectively. Moreover, there was a decline in physical activity behaviors among the older adolescent cohort. In addition, disabilities were negatively associated with sports club membership and MVPA participation for males only. Similar findings have been reported in female populations across 15 European countries [74], and this could be related to the already existing low levels of physical activity participation among females. Nonetheless, several studies have been conducted on PATs to improve the lives of people with impairments [75-78]. Given these study findings, female user-friendly PATs may be a potentially worthwhile future area of research.

### Strengths and Limitations

The data in this study were collected through self-report surveys, and reporting bias from this type of measurement tool is a common limitation in cross-sectional survey-based studies. The data in this study were collected from national representative samples, and such inconsistencies would be typically eradicated by using larger representative samples. Although we attempted to harmonize our data as much as possible, not all items were the same, specifically when translated into the English language. However, the cultural translation, rather than the literal translation, was used in the study to make comparisons possible. This process was carried out by a researcher (KN) with competences in both languages and cultures. Other study limitations are that some residual confounders may be more relevant in one country when compared to the other and therefore were not comparable although stratification by gender and controlling for country, age, family affluence, and disability were included in the adjusted models. Finally, the survey and data collection only gave the options for the respondents to report 3 main types of PATs, and as the market continues to grow, the researchers may have missed some information related to the behaviors from other types of PATs, and the time during which the individual has owned the PATs. The results of this study were cross-sectional, and the length of time that the individuals have been using PATs has not been reported. Increased understanding about the PAT use of young adolescents is needed to not only consider it as a useful tool for promoting physical activity during the adolescent years but also to use it as a part of the daily life at a later stage in adulthood.

### Conclusions

The growing pervasiveness of PAT use across both Finland and Ireland is evident in our study, with similar clustering properties. The association between PAT usage and MVPA provides very useful information for both researchers and practitioners. Evidence from this study highlights the positive physical activity behaviors in adolescents who regularly use and wear PATs, particularly with regards to males. The emergence, pervasiveness, and reducing cost of wearable PATs presents opportunities for researchers to incorporate these into interventions to promote physical activity among young adolescents. Moreover, the application of evidence emerging from physical activity behavior change studies could inform the design and function of future PATs. National efforts in Finland and Ireland should consider using effective dissemination strategies seeking to increase the prevalence of youth gaining access to these wearable devices, while of course acknowledging the feasibility and cost constraints in existence. Advances in technology coupled with reductions in the cost of PATs offer researchers a more viable opportunity to target adolescent-specific physical activity interventions to increase the number of individuals meeting the physical activity guidelines.

## Appendix

Multimedia Appendix 1 Details of the measures in both surveys.

## Abbreviations

CSPPA: Children’s Sport Participation and Physical Activity
FAS: Family Affluence Scale
F-SPA: Finnish School-aged Physical Activity
MVPA: moderate-to-vigorous physical activity
OECD: Organization for Economic Cooperation and Development
OR: odds ratio
PAT: physical activity tracker
