# Supplementary material for: Clusters of Adolescent Physical Activity Tracker Patterns and Their Associations With Physical Activity Behaviors in Finland and Ireland: Cross-Sectional Study
Source: J Med Internet Res. 2020 Sep 1;22(9):e18509. doi: 10.2196/18509 (PMC7492981; doi:10.2196/18509)
Supplement: Multimedia Appendix 1 [file jmir_v22i9e18509_app1.docx]

## Multimedia Appendix 1

Appendix Table 1. Variables that are comparable between CSPPA and F-SPA

| **Variable** | **CSPPA** | **F-SPA** | **Coding** |
| --- | --- | --- | --- |
| Gender | Male  Female  Other | Male  Female | Removal of ‘Other’ |
| Age | Age by Year | Age calculated by month and year of birth and calculation of age, | Grouped into 11, 13, 15y |
| Family Affluence Scale | FAS2 | FAS3 | Relative FAS |
| PA trackers | Apps  Sports watch  HRM  Pedometer  Other,  Use Yes/No  Frequency of use in past week | Frequency of use in the past week | converted pedometer and other data to ‘none’. Apps, SW and HRM into ‘none’, owner’ and ‘user’. |
| Functional Difficulties | CFM | CFM | Grouped into with and without disabilities based on at least one FD with a lot of difficulties |
| MVPA | Two items (past 7 days) and (average 7 days), Cut off from average of two variables (rounded up | Single past 7 days | 0-6 days  7 days |
| Organised sports | Are you currently participating in a club that is organised for a purpose of doing one particular sport or activity (exclude youth club, which may sometimes offer a number of sports)  Yes  No | Are you involved in sports club  Yes competing  Yes, not competing  No, but used too  No and never have | Dichotomised into members and non-members |
| Active Transport | Distance from school  Mode from school  Walking  Bicycling  Bus  Lift by parents | Distance from school  Mode from school  Walking  Bicycling  Bus  Lift by parents | Only those who reported to live within 5km. Active walkers and cyclists. Not active were motor transport |
